# Altered putamen connectivity in patients with neurological post-COVID condition

**DOI:** 10.1093/braincomms/fcaf291

**Published:** 2025-08-09

**Authors:** Lars S Schlenker, Tim J Hartung, Pia Klabunn, Katia Schwichtenberg, Josephine Heine, Lucas Adam, Christiana Franke, Carsten Finke

**Affiliations:** Department of Neurology and Experimental Neurology, Charité—Universitätsmedizin Berlin, Berlin 10117, Germany; Berlin School of Mind and Brain, Humboldt-Universität zu Berlin, Berlin 10117, Germany; Department of Neurology and Experimental Neurology, Charité—Universitätsmedizin Berlin, Berlin 10117, Germany; Department of Neurology and Experimental Neurology, Charité—Universitätsmedizin Berlin, Berlin 10117, Germany; Department of Neurology and Experimental Neurology, Charité—Universitätsmedizin Berlin, Berlin 10117, Germany; Department of Neurology and Experimental Neurology, Charité—Universitätsmedizin Berlin, Berlin 10117, Germany; Department of Neurology and Experimental Neurology, Charité—Universitätsmedizin Berlin, Berlin 10117, Germany; Department of Neurology and Experimental Neurology, Charité—Universitätsmedizin Berlin, Berlin 10117, Germany; Department of Neurology and Experimental Neurology, Charité—Universitätsmedizin Berlin, Berlin 10117, Germany; Berlin School of Mind and Brain, Humboldt-Universität zu Berlin, Berlin 10117, Germany

**Keywords:** post-COVID condition, fatigue, connectome, tractography, neuroimaging

## Abstract

Although the exact aetiology of the post-COVID condition is still under investigation, there is increasing evidence for white matter pathology in patients with persistent cognitive and fatigue symptoms following an infection with SARS-CoV-2. Still, to date there are no studies that investigated the white matter connectome in patients with post-COVID condition. Based on previous findings, we analyzed the structural connectome of these patients, with a focus on the thalamus and basal ganglia. In this cross-sectional study, 43 patients (34 women, 9 men) and 41 (33 women, 8 men) healthy control participants underwent structural MRI, including T1-weighted and diffusion weighted imaging, as well as a comprehensive neuropsychological and psychiatric assessment. The cognitive assessment included verbal and visual long-term memory, working memory, attention, processing speed, executive control and verbal fluency. Fatigue was assessed with the Fatigue Scale for Motor and Cognitive Functions, depression and anxiety were assessed with the Beck Depression Inventory II and the Beck Anxiety Inventory, respectively. MRI data was analyzed using probabilistic tractography, reconstructing 100 million streamlines per participant, to create individual connectomes. Connectome alterations were assessed using graph theory by calculating node strength and betweenness centrality for the thalamus and basal ganglia. We then analyzed group differences in these measures between patients and control participants with the Mann–Whitney-U-test. For significant alterations, we explored associations between graph measures, fatigue and cognition, depression and anxiety using spearman correlations. We identified significantly increased node strength of the putamen (*U* = 589, *p*_FDR_ = 0.036), which was significantly associated with the fatigue severity in patients (*ρ* = 0.33, *P* = 0.045) but not in control participants (*ρ* = 0.11, *P* = 0.509). Betweenness centrality of the putamen was increased in patients with post-COVID condition (*U* = 620, *P* = 0.019) but was not associated with fatigue (*ρ* = 0.07, *P* = 0.685). Neither node strength nor betweenness centrality of the putamen was associated with cognitive performance, depression or anxiety scores. Patients with post-COVID condition exhibit structural connectome alterations that are associated with fatigue severity. Such structural white matter pathology may thus contribute to post-COVID pathophysiology. In addition, putamen connectivity could be a neural correlate of post-COVID fatigue.

## Teaser

White matter pathology is one possible mechanism underlying post-COVID symptoms. Schlenker *et al*. investigated the structural connectome in patients with post-COVID-condition, focusing on the basal ganglia and thalamus. They found altered connectivity of the putamen, which was associated with fatigue but not cognitive dysfunction.

## Graphical Abstract

**Graphical Abstract:**
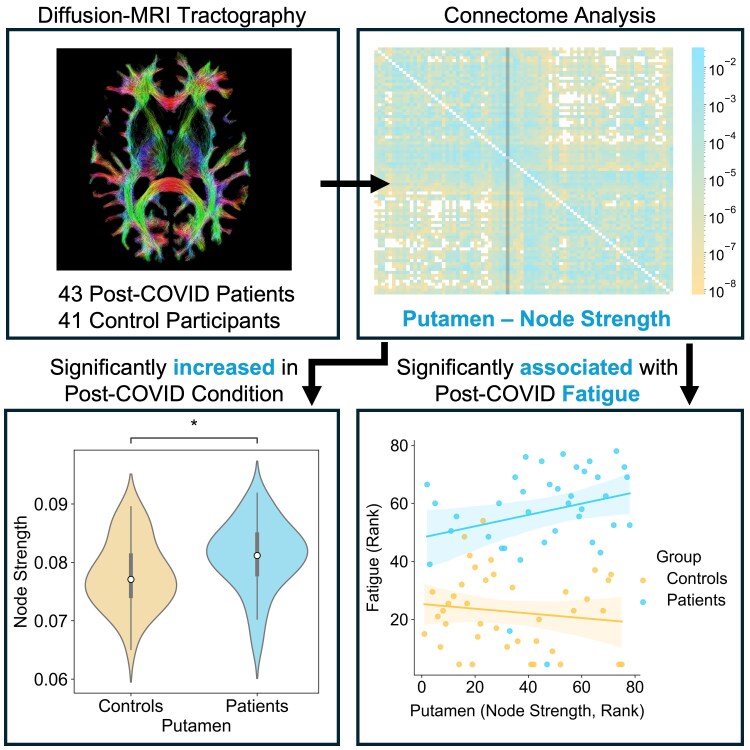

## Introduction

Patients with post-COVID condition (PCC) often present with severe fatigue and cognitive dysfunction^1-5^. PCC is diagnosed if symptoms persist for more than 3 months post infection, affecting up to 3–10% of all COVID-19 (coronavirus disease 2019) patients.^6,7^ While the first cases of PCC were already reported in the early phase of the pandemic,^2^ the exact aetiology of the persisting neurological symptoms still remains unclear.^6^ Ongoing research points towards multiple pathomechanisms, including immune dysregulation with neuroinflammation, endothelial dysfunction, coagulopathy, viral persistence and viral reactivation.^6,8^

Recent evidence shows that immune dysregulation including increased microglial reactivity can result in loss of myelinated axons.^1,9^ Indeed, several neuroimaging studies have observed white matter alterations in patients with PCC, including T2/FLAIR (fluid-attenuated inversion recovery) hyperintensities, decreased perfusion and oxygen levels and changes in diffusion imaging parameters, such as fractional anisotropy (FA), mean diffusivity (MD), axial diffusivity (AD) and radial diffusivity, indicative of altered white matter microstructure^10-23^. T2/FLAIR Hyperintensities have been shown to involve the frontal and parieto-occipital white matter^13^ and the internal capsule.^16^ Likewise, diffusion imaging studies showed widespread alterations in white matter^10,14,15,17,19^ but also more localized alterations in several white matter tracts, including the corona radiata (lower AD,^10^ reduced MD,^21^ and a lower fraction of intracellular water as proxy of axonal density^12^), the external capsule (lower AD^10^) and the corpus callosum (reduced MD,^15^ AD,^15^ FA^17,22^ and also lower fraction of intracellular water^12^). Repeatedly, alterations have also been observed in the internal capsule with lower MD^19^ and lower FA.^17^ Regional findings include altered MD^10^ and altered FA of the thalamus,^11,17^ lower MD of the anterior thalamic radiation^19^ and altered MD of white matter proximal to the putamen and pallidus.^15^ Importantly, white matter alterations have been associated with cognitive deficits and fatigue in patients with PCC.^10,11,13,16,17^

Given the accumulating evidence for widespread white matter damage in PCC, white matter connectivity and accordingly the structural connectome are likely affected by PCC pathology. The connectome describes a comprehensive map of anatomical connections that interlink the neuronal components of the human brain in the form of a connection matrix, offering a framework for network-based analyses of brain structure and function.^24^ While previous studies relied on approaches targeting localized microstructural properties, such as voxel-wise analyses (e.g. TBSS) or regional diffusion metrics, connectome analyses provide a complementary, network-level perspective on white matter organization.

Recently developed tractography algorithms provide a non-invasive way of reconstructing white matter pathways based on diffusion-weighted MRI, thereby enabling the detection of disease-associated connectome alterations. In the past, tractography has been widely used in other neurological disorders with white matter damage and associated cognitive dysfunction and fatigue, including multiple sclerosis and Parkinson's disease.^25,26^ However, despite the growing relevance of understanding neuropsychiatric disorders from a neural network perspective, and the growing evidence for white matter alterations, the structural connectome in patients with PCC has not yet been studied.^27^

For analyzing connectome alterations, graph theory is a widely used approach that translates connectivity data into network metrics, or graph measures, which quantify changes in integration, segregation and hub architecture of the brain.^28^ Two important graph measures are node strength and betweenness centrality. Node strength is one of the most fundamental graph measures, representing the number of connections of a given brain region with all other regions,^25^ and most other graph measures are related to node strength.^28^ Betweenness centrality quantifies how often a region lies on the shortest path between other regions.^28^ While higher node strength indicates how strongly a region is connected to the rest of the network, reflecting its contribution to information integration, betweenness centrality captures the region's role as a connector for communication between other brain regions.^25,28^ Both measures can be used to identify hub regions, which are frequently affected in neurological disorders.^25^

Many of the previously observed white matter alterations are in tracts or regions that are either in close proximity or directly involve the thalamus and basal ganglia^10,11,15,16,18-21^. Similarly, we recently identified structural alterations of the thalamus and basal ganglia that were related to fatigue.^11^ Here, we therefore investigated the structural connectome in a cohort of patients with PCC and healthy controls with a focus on basal ganglia and thalamus structural connectivity. Specifically, we aimed to identify characteristic changes of node strength and betweenness centrality of these brain regions in PCC patients and relate them to individual levels of cognitive dysfunction and fatigue severity. In accordance with previous findings, we hypothesized (i) that PCC patients show reduced structural connectivity of the thalamus and basal ganglia, quantified by lower node strength and betweenness centrality; and (ii) that these changes are associated with cognitive dysfunction and fatigue severity.

## Materials and methods

### Participants

The study received approval from the ethics committee of Charité—Universitätsmedizin Berlin (EA2/007/21) and was conducted in compliance with the Declaration of Helsinki.

Fifty patients with post-COVID condition were recruited from the neurological post-COVID outpatient clinic at Charité—Universitätsmedizin Berlin between April 15 and November 30, 2021. Patients were included if they met the following criteria: (i) a reverse-transcription PCR test confirmed SARS-CoV-2 (severe acute respiratory syndrome coronavirus 2) infection, (ii) postinfectious neurological symptoms for at least 3 months and (iii) no history of neurological or psychiatric disease previous to COVID-19. Additionally, we recruited 50 healthy control participants without a history of neurological or psychiatric disease or known SARS-CoV-2 infection. The control group was matched to the patient group regarding age, sex, and years of education. Six patients and six healthy controls were excluded due to insufficient MRI data quality. In addition, one patient and three controls were excluded for lesions affecting the tractography. Accordingly, 43 patients (34 women, 9 men; median age: 43.16 years) with PCC and 41 healthy controls (33 women, 8 men; median age: 43.73 years) were included in the analysis (Table 1). Five patients and 1 healthy control participant did not provide data on the Fatigue Scale for Motor and Cognitive Functions (FSMC).

**Table 1:** Participant characteristics

	Controls	Patients	*P*-value
Sex	33 women | 8 men	34 women | 9 men	>0.999
Age (years)	43 (32–53)	43 (35–51.5)	0.876
Years of Education	16.5 (15–18)	16.5 (14–18)	0.572
Hospitalized		7 / 43 (16.28%)	
Intensive Care Unit		2 / 43 (4.65%)	
Fatigue (FSMC)	37 (22.75–45.25)	74.53 (15.49)	<0.001
Depression (BDI-II)	3 (0.75–8.25)	16 (10–19.75)	<0.001
Anxiety (BAI)	1 (0–5.5)	13.5 (8–19.5)	<0.001
Acute COVID disease duration (days)		21 (17.5–28)	
Time since infection (days)		243 (204.5–289.5)	

Values are reported with median (interquartile range) if not otherwise indicated. ‘Hospitalized’ and ‘Intensive Care Unit’ indicates the proportion of patients who required hospitalization or intensive care during SARS-CoV-2 infection. FSMC, Fatigue Scale for Motor and Cognitive Functions.

### Neuropsychological and psychiatric assessment

Patients and control participants underwent a comprehensive neuropsychological assessment that included a cognitive screening with the Montreal Cognitive Assessment (MoCA), and the following cognitive tests: German Rey-Auditory Verbal Learning Test (RAVLT)^29^ and Rey-Osterrieth Complex Figure Test (ROCF)^30^ for verbal and visual long-term memory, Digit Span Test forward and backward^31^ for working-memory, German Test of Attentional Performance (TAP)^32^ for attention, Trail-Making Test A (TMT-A) for processing speed and Trail-Making Test B (TMT-B)^33^ as well as the Stroop Word-Color-Interference Test^34^ for executive functions and Regensburger Wortflüssigkeitstest^35^ for verbal fluency. In addition, we assessed anxiety (Beck Anxiety Inventory, BAI), depression (Beck Depression Inventory, BDI-II) and fatigue (FSMC).

### MRI data acquisition

Patient and control MRI data were acquired at the Berlin Centre for Advanced Neuroimaging with a 3T PRISMA scanner and a 64-channel head coil (Siemens, Erlangen, Germany). The MRI protocol included a T1-weigthed scan (3D-MPRAGE (magnetization prepared rapid gradient echo), TR (repetition time) = 1900 ms, TE (echo time) = 2.22 ms, TI (inversion time) = 2100 ms, voxel size 1 × 1 × 1 mm^3^) and a diffusion weighted scan (multiband EPI (echo planar imaging), 98 directions, *b* = [0, 1500, 3000], voxel size 1.5 × 1.5 × 1.5 mm^3^). T1-weighted images were corrected for intensity inhomogeneities during image reconstruction using automated bias field correction.

### MRI data processing and structural connectome generation

T1 images were segmented with Freesurfer 7.2.0.^36^ To delineate cortical and subcortical regions, we parcellated each image using the Desikan–Killiany atlas^37^ implemented in Freesurfer. The basal ganglia were parcellated into the pallidum, putamen and caudate nucleus. Diffusion weighted MRI scans were preprocessed with a standard pipeline using MRtrix3^38^ and FSL.^39^ Preprocessing steps included denoising,^38,40-42^ unringing,^38,43^ and correction for EPI-distortion, Eddy-currents, movement-distortion,^38,44-46^ and bias field correction.^38,47^ After preprocessing, diffusion images were visually inspected for quality assessment, including quality control of the unringed and denoised image and the inspection of remaining (EPI-)distortions. Details are provided in the Supplementary material. Tractography analyses included estimating the fibre orientation distribution in each voxel using multi-shell multi-tissue constrained spherical deconvolution,^48^ followed by an intensity normalization to make the fibre orientation distributions comparable across participants. We segmented the T1-images into grey matter, white matter, and cerebrospinal fluid masks for anatomically constrained tractography. This involves seeding streamlines at the grey-matter-white-matter boundary and rejecting streamlines that end in cerebrospinal fluid or white matter, to increase biological plausibility. We then co-registered the T1-images and the segmented anatomical images to the diffusion weighted images. Finally, we ran the probabilistic second-order Integration over Fibre Orientation Distributions (iFOD2)^49^ tractography algorithm, generating 100 million streamlines per participant and applied the spherical-deconvolution informed filtering of tractograms (SIFT) 2 filter algorithm^50^ to reduce biases and enable biological plausible analysis of tract density by the number of streamlines. In contrast to the original SIFT algorithm, SIFT2 does not remove streamlines but applies a weighting to the connectome. After visual inspection of the tract images—including assessment of anatomically informed streamline orientation, as well as streamline start and termination points (details are provided in the Supplementary material)—we used the co-registered, parcellated T1-images to create structural connectivity matrices for each participant. Resulting connectivity matrices were defined by the number of reconstructed and SIFT-2-weighted streamlines.

### Structural connectivity analysis

We used the Brain Connectivity Toolbox^51^ to analyze structural connectivity with graph theory. Before calculating graph measures, we applied a global L1-normalization within the individual connectivity matrices by dividing each matrix value by the sum of all matrix values, to account for SIFT-2-induced inter-individual variations in the total number of streamlines. Additionally, we merged corresponding regions of the left and right hemispheres within each connectivity matrix, to increase statistical power for subsequent group difference analyses. We then calculated the participant's individual node strength and betweenness centrality for each brain region. Finally, we extracted the graph measures for our regions of interest: the bilateral putamen, caudate, pallidum, and thalamus.

### Statistical analysis

Age, years of education and fatigue scores were not normally distributed. Accordingly, group differences in these sample characteristics were tested with non-parametric Mann–Whitney-U-test. Group differences in sex were tested with the chi-squared test. Likewise, the resulting graph measures were not normally distributed, so we used the Mann–Whitney-U-test to analyze group differences between PCC patients and healthy control participants. To control for multiple comparisons, *P*-values for group differences were corrected using the false discovery rate (FDR) within each graph measure family, with significance set at *P* < 0.05. For graph measures with significant group differences, we further analyzed associations between the measure and the fatigue score using the spearman correlation coefficient. Exploratory analyses were additionally conducted to assess associations with neuropsychological test scores (raw values), depression (BDI-II) and anxiety (BAI) scores, restricted to graph measures exhibiting significant group differences. Correlation analyses were conducted separately for patients and control participants, to identify clinically relevant associations. Correlation analyses for associations with fatigue were restricted to the 78 participants who had completed FSMC data. For significant associations with the FSMC, we re-evaluated any previously observed group difference in the restricted sample to ensure that the result was not driven by the six participants lacking FSMC data.

## Results

### Sample characteristics

Patients with PCC and control participants did not differ significantly regarding sex, age or years of education. The patient showed significantly increased fatigue, depression and anxiety scores in comparison to the control participants (Table 1). Additionally, patients exhibited significantly reduced cognitive performance relative to control participants in all tested cognitive domains, except working memory (digit span backwards, Table 2). Here we report relevant scores for each cognitive domain. Detailed results from all tests are provided in the Supplementary material.

**Table 2:** Neuropsychological group differences

Neuropsychological test	Cognitive domain	Controls (median)	Patients (median)	Mann– Whitney *U*	*P*-value	*P*-value Adj.	Rank- Biserial *r*
MoCa	Global Cognition	28	27	1216.5	**0**.002**	**0**.004**	0.33
RAVLT—Delayed Recall	Verbal Memory	14	11	1263.5	**0**.001**	**0**.002**	0.37
ROCF—Delayed Recall	Visual Memory	26	19	1242.0	**0**.001**	**0**.003**	0.35
Digit Span Backwards	Working Memory	7	6	1062.5	0.100	0.100	0.18
TAP—Tonic Alertness	Attention	246	275	373.5	**<0**.001***	**<0**.001***	−0.5
TMT A	Processing Speed	25	33	587.5	**0**.009**	**0**.012*	−0.29
TMT B	Executive Functioning^a^	51	59	597.5	**0**.011*	**0**.014*	−0.28
Word-Color-Interference	Executive Functioning^b^	101	114	488.5	**<0**.001***	**0**.002**	−0.38
Fluency Test: Animals	Verbal Fluency	27	24	1237.0	**0**.001**	**0**.003**	0.35

Group differences between patients and controls for the neuropsychological test scores. *P < 0.05, **P < 0.01, ***P < 0.001. Bold *P*-values indicate a significant group difference. MoCa, Montreal Cognitive Assessment; RAVLT, German Rey-Auditory Verbal Learning Test; ROCF, Rey-Osterrieth Complex Figure Test; TAP, German Test of Attentional Performance; TMT, Trail Making Test.

^a^Set-shifting.

^b^Inhibition.

### Region-of-interest analyses

**Figure 1:**
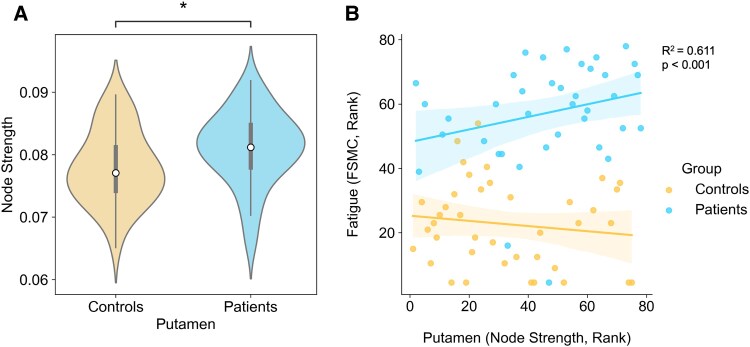
**Between-group differences of Putamen node strength: (A): group difference (Mann–Whitney U-test) in node strength of the putamen (*N* = 84).** Patients with PCC exhibited significantly higher node strength of the putamen compared with healthy controls. *p_FDR_ < 0.05. (**B**) Spearman Correlation between node strength of the putamen and the fatigue symptoms (FSMC) per group (*N* = 78). Each data point refers to a participant's score in nodes strength of the putamen and the corresponding FSMC score. Node strength of the putamen only correlated with fatigue in patients with PCC (*ρ* = 0.33, *P* = 0.045), with a trend-level group interaction effect (multiple linear regression analysis), *t* = 1.81, *P* = 0.074.

Results from the region-of-interest analyses are summarized in Tables 3 and 4. We found a significantly higher node strength of the putamen in PCC patients compared with control participants (Fig. 1A). The group difference remained significant when the analysis was restricted to the 78 participants with complete FSMC and tractography data (Mann–Whitney *U* = 496, *P* = 0.008). In the patient group, higher node strength of the putamen was significantly associated with more severe fatigue, *ρ* = 0.33, *P* = 0.045. In contrast, there was no significant correlation between the node strength of the putamen and fatigue in the healthy control group, indicative for a group interaction effect (Fig. 1B). To test this effect, we ran a multiple linear regression with an interaction term (group and node strength of the putamen), which indicated a trend-level interaction effect, *t* = 1.81, *P* = 0.074.

**Table 3:** Connectome analysis—node strength

ROI	Controls (median)	Patients (median)	Mann–Whitney *U*	*P*-value	*P*-value Adj.
Caudate	0.0790	0.0755	940	0.604	0.604
Putamen	0.0771	0.0812	589	**0**.009**	**0**.036*
Pallidum	0.0420	0.0407	978	0.390	0.520
Thalamus	0.1093	0.1064	1000	0.291	0.520

Results from the connectome analyses. **P < 0.01, *P < 0.05. *P*-value Adj. = False-Discovery Rate Adjusted. Bold *P*-values indicate a significant group difference.

**Table 4:** Connectome analysis—betweenness centrality

ROI	Controls (median)	Patients (median)	Mann–Whitney *U*	*P*-value	*P*-value Adj.
Caudate	22	28	901	0.863	0.863
Putamen	80	54	620	**0**.019*	0.078
Pallidum	0	0	902.5	0.702	0.863
Thalamus	354	352	934	0.641	0.863

Results from the connectome analyses. **P < 0.01, *P < 0.05. *P*-value Adj. = False-Discovery Rate Adjusted. Bold *P*-values indicate a significant group difference.

At the uncorrected threshold, the putamen showed higher betweenness centrality in PCC patients than in controls (Table 4). This difference did not survive FDR correction. Betweenness centrality was not significantly associated with fatigue severity. Neither node strength nor betweenness centrality of the putamen was significantly associated with any of the assessed cognitive scores (Tables 5 and 6), depression (BDI-II; node strength: *ρ* = 0.19, *P* = 0.244; betweenness centrality: *ρ* = −0.04, *P* = 0.836) or anxiety (BAI; node strength: *ρ* = 0.19, *P* = 0.244; betweenness centrality: *ρ* = −0.05, *P* = 0.761) in patients with PCC.

**Table 5:** Correlations—Putamen node strength with cognition

Neuropsychological test	Cognitive domain	Spearman *ρ*	*P*-value
MoCA	Global Cognition	0.143	0.361
RAVLT—Delayed Recall	Verbal Memory	−0.087	0.578
ROCF—Delayed Recall	Visual Memory	−0.007	0.967
Digit Span Backwards	Working Memory	0.209	0.178
TAP—Tonic Alertness	Attention	0.197	0.206
TMT A	Processing Speed	−0.133	0.395
TMT B	Executive Functioning^a^	−0.101	0.521
Word-Color-Interference	Executive Functioning^b^	−0.066	0.674
Fluency Test—Animals	Verbal Fluency	0.158	0.313

Spearman correlation between nodes strength of the putamen and neuropsychological test scores. MoCa, Montreal Cognitive Assessment; RAVLT, German Rey-Auditory Verbal Learning Test; ROCF, Rey-Osterrieth Complex Figure Test; TAP, German Test of Attentional Performance; TMT, Trail Making Test.

^a^Set-shifting.

^b^Inhibition.

**Table 6:** Correlations—Putamen betweenness centrality with cognition

Neuropsychological test	Cognitive domain	Spearman *ρ*	*P*-value
MoCA	Global Cognition	−0.143	0.360
RAVLT—Delayed Recall	Verbal Memory	−0.002	0.990
ROCF—Delayed Recall	Visual Memory	0.061	0.699
Digit Span Backwards	Working Memory	0.044	0.781
TAP—Tonic Alertness	Attention	0.280	0.069
TMT A	Processing Speed	−0.135	0.389
TMT B	Executive Functioning^a^	−0.226	0.146
Word-Color-Interference	Executive Functioning^b^	−0.033	0.836
Fluency Test—Animals	Verbal Fluency	0.044	0.781

Spearman correlation between betweenness centrality of the putamen and neuropsychological test scores. MoCa, Montreal Cognitive Assessment; RAVLT, German Rey-Auditory Verbal Learning Test; ROCF, Rey-Osterrieth Complex Figure Test; TAP, German Test of Attentional Performance; TMT, Trail Making Test.

^a^Set-shifting.

^b^Inhibition.

## Discussion

In this study, we applied state-of-the-art tractography to identify white matter connectivity changes and potential clinical associations in patients with PCC. We hypothesized that patients with PCC would show decreased structural connectivity of the thalamus, caudate, pallidum and putamen. In contrast, we found increased structural connectivity (i.e. increased node strength) of the putamen in PCC patients in comparison to healthy control participants. Moreover, increased node strength was associated with more severe fatigue in patients, but not in control participants, providing evidence for a PCC-related white matter dysfunction underlying fatigue severity. Compared with our previous work, which focused on regional diffusion tensor imaging (DTI) and volumetric alterations within the basal ganglia and thalamus,^11^ the current study extends these findings by examining the connectivity profile of the same regions of interest using tractography and network-based measures. We thereby complement previous regional findings with evidence for large-scale network-level reorganization.

### Findings in relation to previous research

Neuroimaging studies have consistently reported both structural and functional brain alterations in patients with PCC, particularly in relation to fatigue and cognitive dysfunction. Affected regions include the thalamus and putamen, as well as cortical areas, the cerebellum, brainstem and limbic structures such as the hippocampus and parahippocampal gyrus^23,52-54.^ Structural MRI studies have primarily shown reduced grey matter volumes^54^ in these regions, although some reported increased volume in the basal ganglia and thalamus.^55,56^ Diffusion imaging studies, including DTI and more recently developed diffusion microstructural imaging, have similarly identified widespread white matter alterations.^10,11,23,54^ Functional connectivity studies further support these findings, revealing altered connectivity patterns in the brainstem, thalamus, cerebellum and limbic regions such as the cingulate and olfactory cortex.^15,52-54^

Across diffusion imaging studies, white matter alterations appear to be a robust finding in PCC, affecting multiple tracts and brain regions^10-23^. Consistently reported alterations include the corpus callosum,^12,15,17,22,23^ the corona radiata,^10,12,19,21^ the internal capsule^16,17,19,23^ and the thalamic radiation.^19,54^ Nonetheless, these studies primarily relied on approaches targeting localized microstructural properties, such as voxel-wise analyses (e.g. TBSS) or regional diffusion metrics, thereby providing insights into local alterations but offering limited understanding of network-level disruptions.

To date, studies explicitly investigating the structural connectome using tractography-based approaches in PCC are lacking. Our study therefore complements existing literature by extending previous local findings to the connectome level, revealing altered putamen connectivity and its association with fatigue severity. This adds a novel system-level perspective to the existing evidence of white matter pathology in PCC.

Among the various region implicated in PCC, the putamen has emerged as particularly relevant structure due to its central role in fatigue pathophysiology. Recent studies have identified the putamen as one of the key areas linked to fatigue pathophysiology in PCC, showing volumetric changes and deviations in diffusion parameters that correlate with fatigue severity^11,23,55-57^. Indeed, the association between altered putamen connectivity and fatigue is plausible given previous imaging findings in other neuroimmunological diseases and in aging. In multiple sclerosis, functional and structural connectivity of the striatum, including the putamen, are associated with subjective and objective measures of cognitive fatigue^58-62^. Additionally, altered microstructural properties of the putamen have been linked to fatigue in multiple sclerosis.^59^ In older healthy adults, smaller basal ganglia volume was associated with worse fatigue, with the putamen exhibiting the strongest correlation of the investigated basal ganglia structures.^63^ In another study with healthy individuals, functional connectivity analyses have been used to delineate a ‘fatigue-network’, with the striatum, including the putamen, as central hub.^64^ In the current study, we observed network alterations of the putamen in patients with PCC that were associated with fatigue severity, adding to converging evidence from neuroimmunological disorders, aging research and healthy populations highlighting the putamen's involvement in fatigue pathophysiology.

While these findings implicate that the putamen is central in fatigue, they also raise the question of how the putamen is exactly involved in the pathophysiology of fatigue. Anatomically, the putamen is part of the basal ganglia, which form a central part of several dopaminergic striato-cortical circuits, with strong connections to the prefrontal cortex.^65,66^ They play a crucial role not only in motor control but also in executive functions and motivation, possibly regulating energy expenditure.^65,67^ In particular, dysfunction of the non-motor circuits, affecting primarily motivational processes, is thought to contribute to fatigue symptoms.^65^ More recently, this hypothesis has been extended, with evidence for a dopamine imbalance in the prefrontal cortex and the striatum, as a consequence of damage to dopaminergic neurons or pathways.^66^ Interestingly, recent studies have shown a detrimental effect of inflammatory cytokines on dopaminergic neurons,^66,68^ with neuroinflammation possibly playing a role in the emergence of PCC.^6,8^

### Interpretation of increased connectivity

#### Compensatory connectivity

In our study, we found increased structural connectivity in patients with PCC. While decreased connectivity is more frequently reported in neurological disorders there is also evidence of increased connectivity,^69-73^ particularly in subcortical regions such as the putamen, often accompanied by volume reductions.^70,72,73^ This pattern was similarly observed in our cohort. Such increased connectivity is commonly interpreted as compensatory response to neural injury, aiming to maintain network function^74-76^. Although most evidence stems from functional imaging studies, structural and functional connectivity are closely related.^77,78^ Thus, our findings may reflect an initial compensatory mechanism following SARS-CoV-2 infection. However, the positive association between increased connectivity and fatigue challenges the notion of successful compensation.

#### Maladaptive connectivity

In contrast, increased connectivity might be part of the underlying pathology of PCC, i.e. directly or indirectly caused by SARS-CoV-2 infection, leading to the observed fatigue symptoms. With more streamlines passing through the putamen, these new connections could be either futile or the general neural information transfer might become less efficient. In line with this, it has been reported that greater modularity (i.e. strongly connected hubs) comes at the cost of global information integration in connectomes.^27^ Furthermore, higher connectivity is metabolically demanding^74,76^ and might therefore be maladaptive in patients with PCC. The direction of the positive association with fatigue—that was only present in patients and not in healthy controls—is in line with this hypothesis. However, further research ideally on longitudinal cohorts, is required to elucidate the underlying mechanisms.

#### Vulnerability

Another alternative explanation is that the observed connectivity pattern in patients was already present before the infection. In our previous study of the same cohort, we observed volume reduction and surface deformations of the putamen.^11^ If we assume that patients had a stronger connectivity of the putamen before the infection, indicative of a greater relevance for information processing (hub region), then any local damage to the very same region would have a much stronger negative effect compared with regions with a low connectivity profile. The observed increased connectivity might be the result of initially persisting white matter tracts that only later deteriorate. A greater premorbid connectivity together with COVID associated damage of the putamen would thus represent a vulnerability for the development of PCC.

This hypothesis is in line with findings on computationally simulated attacks (corresponding to neurological pathology) targeting highly connected and central nodes, including the putamen, in structural connectomes.^75^ Attacks on these nodes resulted in a 3-fold greater reduction in overall communication efficiency than attacks targeting nodes with lower connectivity and centrality.^75^

### Clinical implications

Our findings of aberrant putamen connectivity in patients with PCC and its association with fatigue severity highlight the potential of structural connectome analyses as complementary tool in clinical assessment. In contrast to conventional MRI, connectome-based approaches can detect subtle network alterations that may underlie persistent symptoms such as fatigue, thereby providing a pathophysiological correlate for clinical manifestations^25,27,53,79-82^. With recent advances in imaging acquisition, tractography algorithms and high-performance computing, connectome analyses are becoming increasingly accessible for clinical applications. In this context, detection of altered putamen connectivity may serve as an objective imaging biomarker to support the differential diagnosis of PCC-related fatigue and to monitor disease progression or response to targeted interventions addressing fatigue symptoms.

### Limitations

There are some limitations of our study that should be noted. This study employed a monocentric recruitment approach. Future multicenter studies will facilitate the recruitment of larger samples and enhance the generalizability of findings. Other limitations were the small sample size and the lack of a control group consisting of individuals who were infected with SARS-CoV-2 but did not develop post-COVID condition. Future studies should replicate our findings in larger cohorts and include additional control groups—particularly individuals with prior SARS-CoV-2 infection who remained asymptomatic or recovered without persistent neurological symptoms—to better isolate the effects specific to post-COVID condition. Finally, tractography is an evolving method with some limitations, including the generation of false-positive connections and diverse biases.^25^ We addressed these issues by applying state-of-the-art methods, including probabilistic tractography with multi-shell multi-tissue constrained spherical deconvolution, anatomical constraints, and a SIFT-2 filter algorithm, to maximize biological plausibility. Moreover, we used high-performance computing to enable the reconstruction of a massive number of 100 million streamlines per participant, optimizing the signal-to-noise ratio. Regarding the rapid evolution of tractography, newly developed and further optimized algorithms should be applied in the future to replicate prior tractography studies, including ours.

## Conclusion

Patients with PCC show altered structural connectivity of the putamen associated with fatigue severity. These findings suggest a disease-related role of these alterations, providing new insights in the pathology of PCC-associated fatigue and corroborating the key role of the putamen in the pathophysiology of PCC.

## Supplementary Material

fcaf291_Supplementary_Data

## Data Availability

Data supporting the findings of this study are available from the corresponding author upon reasonable request. The code supporting the findings of this study is publicly available in the following repository: https://github.com/larsschlenker/tractography_pipeline.git.
