# Correction to: Altered putamen connectivity in patients with neurological post-COVID condition

**DOI:** 10.1093/braincomms/fcag209

**Published:** 2026-07-17

This is a correction to: Lars S Schlenker, Tim J Hartung, Pia Klabunn, Katia Schwichtenberg, Josephine Heine, Lucas Adam, Christiana Franke, Carsten Finke, Altered putamen connectivity in patients with neurological post-COVID condition, *Brain Communications*, Volume 7, Issue 4, 2025, fcaf291, https://doi.org/10.1093/braincomms/fcaf291

The originally published version of this manuscript has been corrected to remove preliminary data from a spatial navigation test (Virtual Environments Navigation Assessment for young and middle-aged adults; VIENNA YOUNG), that had not yet undergone full quality control.

This data has now been removed from the article's Abstract, Material and Methods section, and Results section, as well as from Tables 2, 5, and 6 and the supplementary data file.

Additionally, the following reference, originally listed in the article as reference 36, has been removed:

Rekers S, Finke C. Translating spatial navigation evaluation from experimental to clinical settings: The virtual environments navigation assessment (VIENNA). Behav Res Methods. 2024;56(3): 2033-2048.

All references following the removed reference have been renumbered.

All other data, including the imaging analyses and standardized cognitive assessments, have been re-checked by the authors who confirmed they were accurate.

