# Supplementary material for: Altered putamen connectivity in patients with neurological post-COVID condition
Source: Brain Commun. 2025 Aug 9;7(4):fcaf291. doi: 10.1093/braincomms/fcaf291 (PMC12351164; doi:10.1093/braincomms/fcaf291)
Supplement: fcaf291_Supplementary_Data [file fcaf291_supplementary_data.docx]

Supplementary material

DWI Preprocessing Tools

MRtrix3:

- Denoising: dwidenoise^1–4^
- Unringing: mrdegibbs^4,5^
- Motion and distortion correction: dwifslpreproc^4,6–8^
- Bias field correction: dwibiascorrect^4,9^

FSL:

- Brain extraction: BET2^10^

Tractography Tools (Parameters)

MRtrix3:

- Response function estimation: dwi2response (dhollander)^11^
- Fiber orientation distribution estimation: dwi2fod (msmt_csd)^12^
- Intensity normalization: mtnormalize^13^
- 5-tissue-type image: 5ttgen^14^ (fsl^10^)
- Probabilistic tractography: tckgen^4,15^ (act, backtrack, seed dynamic, select 100M)
- SIFT2: tcksift2^16^ (act)
- Connectome construction: tck2connectome (symmetric, zero_diagonal, tck_weights_in)^17^

Visual Quality Inspection Steps

- After denoising, we calculated the difference between the raw and the denoised images and visualized the residual.
- After unringing, we calculated the difference between the denoised image and the unringed image and visualized the residual
- After motion and distortion correction, we overlaid the preprocessed image with the input image and assessed remaining (EPI-)distortions
- After bias field correction, we visualized the bias field, followed by brain mask estimation with the bias field corrected and the uncorrected image to assess whether the bias field correction improved the brain mask estimation
- After response function estimation, we visually inspected the response functions for white matter, gray matter and cerebrospinal fluid
- After estimating the fiber orientation distribution, we visualized the distribution in voxels within anatomical regions with known orientation distributions, such as the corpus callosum, cingulum, and corticospinal tract
- After co-registration of T1 to the DWI image, we overlaid both images and assessed any deviations
- After preparing a gray-matter/white-matter boundary as streamline see region, we visually inspected the boundary by overlaying the image with the T1 image and assess any deviations
- After running the probabilistic tractography algorithm, we visually inspected a subset (100.000 streamlines) of the reconstructed streamlines by overlaying them with the T1 image and the DWI image and assessed streamline orientation within anatomical regions with known streamline orientations (corpus callosum, cingulum and corticospinal tract) and assessed any start or termination within gray matter or cerebrospinal fluid.

Neuropsychological Data

Supplementary Table 1: Neuropsychological Group Differences

| Neuropsychological Test | Mann-Whitney U | P-value | P-Value Adj. | Rank- Biserial *r* |
| --- | --- | --- | --- | --- |
| MoCa | 1216.5 | **0.002**** | **0.006** | 0.33 |
| VLMT 1 | 1066.0 | 0.095 | 0.11 | 0.18 |
| VLMT 5 | 1205.0 | **0.002**** | **0.006**** | 0.32 |
| VLMT sum | 1132.0 | **0.025*** | **0.034*** | 0.24 |
| VLMT 7 | 1263.5 | **0.001**** | **0.003**** | 0.37 |
| VLMT recognition | 1113.0 | **0.027*** | **0.034*** | 0.23 |
| VLMT 5 - 7 | 594.5 | **0.009**** | **0.016*** | -0.28 |
| ROCF immediate | 1136.5 | **0.023*** | **0.033*** | 0.25 |
| ROCF delay | 1242.0 | **0.001**** | **0.004**** | 0.35 |
| Digit span forward | 993.0 | 0.316 | 0.316 | 0.11 |
| Digit span backward | 1062.5 | 0.1 | 0.11 | 0.18 |
| TAP alertness tonic | 373.5 | **< 0.001***** | **< 0.001***** | -0.5 |
| TAP alertness phasic | 575.5 | **0.006**** | **0.013*** | -0.3 |
| TAP selective attention | 610.5 | **0.015*** | **0.024*** | -0.26 |
| TAP dual auditory | 510.0 | **0.001**** | **0.004**** | -0.36 |
| TAP dual visual | 519.5 | **0.001**** | **0.004**** | -0.35 |
| TMT A | 587.5 | **0.009**** | **0.016*** | -0.29 |
| TMT B | 597.5 | **0.011*** | **0.018*** | -0.28 |
| Word-Color-Interference | 488.5 | **< 0.001***** | **0.003**** | -0.38 |
| Fluency phonetic: | 1537.5 | **< 0.001***** | **< 0.001***** | 0.64 |
| Fluency semantic: animals | 1237.0 | **0.001**** | **0.004**** | 0.35 |

Supplementary Table 1: Group differences between patients and controls for the neuropsychological test scores. *: p < 0.05, **: p < 0.01, ***p < 0.001, MoCa = Montreal Cognitive Assessment, VLMT = German Rey-Auditory Verbal Learning Test, ROCF = Rey-Osterrieth Complex Figure Test, TAP = German Test of Attentional Performance, TMT = Trail Making Test. Bold test names and bold p-values indicate a significant group difference.

Supplementary Table 2: Correlations between putamen node strength and cognition

| Neuropsychological Test | Spearman ρ | P-value |
| --- | --- | --- |
| MoCA | 0.143 | 0.361 |
| VLMT 1 | 0.058 | 0.711 |
| VLMT 5 | -0.143 | 0.362 |
| VLMT sum | -0.056 | 0.721 |
| VLMT 7 | -0.087 | 0.578 |
| VLMT recognition | 0.010 | 0.951 |
| VLMT 5-7 | 0.006 | 0.968 |
| ROCF immediate | -0.061 | 0.696 |
| ROCF delay | -0.007 | 0.967 |
| Digits forward | 0.089 | 0.572 |
| Digits backward | 0.209 | 0.178 |
| TAP alertness tonic | 0.197 | 0.206 |
| TAP alertness phasic | 0.159 | 0.310 |
| TAP selective | 0.155 | 0.319 |
| TAP dual auditory | 0.028 | 0.861 |
| TAP dual visual | 0.110 | 0.481 |
| TMT A | -0.133 | 0.395 |
| TMT B | -0.101 | 0.521 |
| World-Color-Interference | -0.066 | 0.674 |
| Fluency phonetic: s | 0.228 | 0.142 |
| Fluency semantic: animals | 0.158 | 0.313 |

Supplementary Table 2: Spearman correlation between nodes strength of the putamen and neuropsychological test scores. MoCa = Montreal Cognitive Assessment, VLMT = German Rey-Auditory Verbal Learning Test, ROCF = Rey-Osterrieth Complex Figure Test, TAP = German Test of Attentional Performance, TMT = Trail Making Test.

Supplementary Table 3: Correlations between putamen betweenness centrality and cognition

| Neuropsychological Test | Spearman ρ | P-value |
| --- | --- | --- |
| MoCA | -0.143 | 0.360 |
| VLMT 1 | -0.024 | 0.880 |
| VLMT 5 | -0.020 | 0.897 |
| VLMT Sum | 0.018 | 0.911 |
| VLMT 7 | -0.002 | 0.990 |
| VLMT recognition | 0.242 | 0.117 |
| VLMT 5 - 7 | -0.019 | 0.903 |
| ROCF immediate | -0.028 | 0.857 |
| ROCF delay | 0.061 | 0.699 |
| Digits forward | -0.162 | 0.300 |
| Digits backward | 0.044 | 0.781 |
| TAP alertness tonic | 0.280 | 0.069 |
| TAP alertness phasic | 0.207 | 0.183 |
| TAP selective | 0.096 | 0.541 |
| TAP dual auditory | -0.236 | 0.128 |
| TAP dual visual | -0.093 | 0.554 |
| TMT A | -0.135 | 0.389 |
| TMT B | -0.226 | 0.146 |
| Word-Color-Interference | -0.033 | 0.836 |
| Fluency phonetic: s | -0.140 | 0.369 |
| Fluency semantic: animals | 0.044 | 0.781 |

Supplementary Table 3: Spearman correlation between betweenness centrality of the putamen and neuropsychological test scores. MoCa = Montreal Cognitive Assessment, VLMT = German Rey-Auditory Verbal Learning Test, ROCF = Rey-Osterrieth Complex Figure Test, TAP = German Test of Attentional Performance, TMT = Trail Making Test.**References:**

1. Veraart J, Novikov DS, Christiaens D, Ades-aron B, Sijbers J, Fieremans E. Denoising of diffusion MRI using random matrix theory. *NeuroImage*. 2016;142:394-406. doi:10.1016/j.neuroimage.2016.08.016

2. Veraart J, Fieremans E, Novikov DS. Diffusion MRI noise mapping using random matrix theory. *Magn Reson Med*. 2016;76(5):1582-1593. doi:10.1002/mrm.26059

3. Cordero-Grande L, Christiaens D, Hutter J, Price AN, Hajnal JV. Complex diffusion-weighted image estimation via matrix recovery under general noise models. *NeuroImage*. 2019;200:391-404. doi:10.1016/j.neuroimage.2019.06.039

4. Tournier JD, Smith R, Raffelt D, et al. MRtrix3: A fast, flexible and open software framework for medical image processing and visualisation. *NeuroImage*. 2019;202:116137. doi:10.1016/j.neuroimage.2019.116137

5. Kellner E, Dhital B, Kiselev VG, Reisert M. Gibbs-ringing artifact removal based on local subvoxel-shifts. *Magn Reson Med*. 2016;76(5):1574-1581. doi:10.1002/mrm.26054

6. Andersson JLR, Sotiropoulos SN. An integrated approach to correction for off-resonance effects and subject movement in diffusion MR imaging. *NeuroImage*. 2016;125:1063-1078. doi:10.1016/j.neuroimage.2015.10.019

7. Smith SM, Jenkinson M, Woolrich MW, et al. Advances in functional and structural MR image analysis and implementation as FSL. *NeuroImage*. 2004;23:S208-S219. doi:10.1016/j.neuroimage.2004.07.051

8. Andersson JLR, Skare S, Ashburner J. How to correct susceptibility distortions in spin-echo echo-planar images: application to diffusion tensor imaging. *NeuroImage*. 2003;20(2):870-888. doi:10.1016/S1053-8119(03)00336-7

9. Tustison NJ, Avants BB, Cook PA, et al. N4ITK: Improved N3 Bias Correction. *IEEE Trans Med Imaging*. 2010;29(6):1310-1320. doi:10.1109/TMI.2010.2046908

10. Jenkinson M, Beckmann CF, Behrens TEJ, Woolrich MW, Smith SM. FSL. *NeuroImage*. 2012;62(2):782-790. doi:10.1016/j.neuroimage.2011.09.015

11. Dhollander T, Raffelt D, Connelly A. Unsupervised 3-tissue response function estimation from single-shell or multi-shell diffusion MR data without a co-registered T1 image. In: ; 2016.

12. Jeurissen B, Tournier JD, Dhollander T, Connelly A, Sijbers J. Multi-tissue constrained spherical deconvolution for improved analysis of multi-shell diffusion MRI data. *NeuroImage*. 2014;103:411-426. doi:10.1016/j.neuroimage.2014.07.061

13. Raffelt D, Dhollander T, Tournier JD, et al. Bias Field Correction and Intensity Normalisation for Quantitative Analysis of Apparent Fibre Density. In: Vol 25. ; 2017:3541.

14. Smith RE, Tournier JD, Calamante F, Connelly A. Anatomically-constrained tractography: Improved diffusion MRI streamlines tractography through effective use of anatomical information. *NeuroImage*. 2012;62(3):1924-1938. doi:10.1016/j.neuroimage.2012.06.005

15. Tournier JD, Calamante F, Connelly A. Improved probabilistic streamlines tractography by 2nd order integration over fibre orientation distributions. *Proc Int Soc Magn Reson Med*. Published online 2010:1670.

16. Smith RE, Tournier JD, Calamante F, Connelly A. SIFT2: Enabling dense quantitative assessment of brain white matter connectivity using streamlines tractography. *NeuroImage*. 2015;119:338-351. doi:10.1016/j.neuroimage.2015.06.092

17. Smith RE, Tournier JD, Calamante F, Connelly A. The effects of SIFT on the reproducibility and biological accuracy of the structural connectome. *NeuroImage*. 2015;104:253-265. doi:10.1016/j.neuroimage.2014.10.004
